# Effect of Dietary Forage to Concentrate Ratios on Dynamic Profile Changes and Interactions of Ruminal Microbiota and Metabolites in Holstein Heifers

**DOI:** 10.3389/fmicb.2017.02206

**Published:** 2017-11-09

**Authors:** Jun Zhang, Haitao Shi, Yajing Wang, Shengli Li, Zhijun Cao, Shoukun Ji, Yuan He, Hongtao Zhang

**Affiliations:** ^1^State Key Laboratory of Animal Nutrition, Beijing Engineering Technology Research Center of Raw Milk Quality and Safety Control, College of Animal Science and Technology, China Agricultural University, Beijing, China; ^2^Department of Animal and Poultry Science, University of Saskatchewan, Saskatoon, SK, Canada

**Keywords:** rumen microbiome, rumen metabolomics, forage to concentrate ratio, high concentrate, heifer

## Abstract

A better understanding of global ruminal microbiota and metabolites under extensive feeding conditions is a prerequisite for optimizing rumen function and improving ruminant feed efficiency. Furthermore, the gap between the information on the ruminal microbiota and metabolites needs to be bridged. The aim of this study was to investigate the effects of a wide range of forage to concentrate ratios (F:C) on changes and interactions of ruminal microbiota and metabolites. Four diets with different F:C (80:20, 60:40, 40:60, and 20:80) were limit-fed to 24 Holstein heifers, and Illumina MiSeq sequencing and gas chromatography time-of-flight/mass spectrometry were used to investigate the profile changes of the ruminal microbes and metabolites, and the interaction between them. The predominant bacterial phyla in the rumen were *Bacteroidetes* (57.2 ± 2.6%) and *Firmicutes* (26.8 ± 1.6%), and the predominant anaerobic fungi were Neocallimastigomycota (64.3 ± 3.8%) and Ascomycota (22.6 ± 2.4%). In total, 44, 9, 25, and 2 genera, respectively, were identified as the core rumen bacteria, ciliate protozoa, anaerobic fungi, and archaea communities across all samples. An increased concentrate level linearly decreased the relative abundance of cellulolytic bacteria and ciliates, namely *Fibrobacter, Succinimonas, Polyplastron*, and *Ostracodinium* (*q* < 0.05), and linearly increased the relative abundance of *Entodinium* (*q* = 0.04), which is a non-fibrous carbohydrate degrader. Dietary F:C had no effect on the communities of anaerobic fungi and archaea. Rumen metabolomics analysis revealed that ruminal amino acids, lipids, organic acids, and carbohydrates were altered significantly by altering the dietary F:C. With increasing dietary concentrate levels, the proportions of propionate and butyrate linearly increased in the rumen (*P* ≤ 0.01). Correlation analysis revealed that there was some utilization relationship or productive association between candidate metabolites and affected microbe groups. This study provides a better understanding of ruminal microbiota and metabolites under a wide range of dietary F:C, which could further reveal integrative information of rumen function and lead to an improvement in ruminant production.

## Introduction

The rumen is a complex microbial ecosystem and is inhabited by a high density of resident microbiota, with bacteria, ciliate protozoa, anaerobic fungi, and archaea involved (Hobson and Stewart, 1997). Rumen microorganisms play important roles in the degradation of feedstuffs and the production of volatile fatty acids (VFAs), lactate, amino acids, lipids, and hydrogen, which are crucial to the maintenance, growth, and production performance of ruminants (Shabat et al., 2016). Due to the complexity of the rumen ecosystem, a better understanding of the ruminal microbiota under extensive feeding conditions is a necessary prerequisite for the manipulation of the rumen microbiota to optimize rumen function and improve feed efficiency (Jami and Mizrahi, 2012; Bannink et al., 2016).

Although the effect of different forage to concentrate ratios (F:C) on rumen microbiota has been investigated, only two or three levels of F:C were used, which does not provide a dataset that is large enough to understand the microbiota variation (Saleem et al., 2012; Mao et al., 2016; Hua et al., 2017). When a high level of concentrate (usually about 50–65% concentrate on a dry matter basis) is used in diets in a sudden change, dairy cows are at a high risk of suffering from subacute ruminal acidosis (SARA), which results in a high risk of chronic inflammation, lameness, mastitis, and laminitis (Zebeli and Metzler-Zebeli, 2012; Mao et al., 2016; Hua et al., 2017). However, limit-feeding dairy heifers with high-concentrate diets was proposed as a potential strategy to improve feed efficiency in dairy farms (Zanton and Heinrichs, 2007, 2009, 2016) and can serve as an excellent model for examining the effects of a wide range of F:C on the ruminal microbiota. In most cases, limit-feeding in heifers refers to heifers that are fed with high-concentrate diets to meet, but not exceed, energy requirements for an optimal level of average daily gain (Zanton and Heinrichs, 2009, 2016). The importance of the microbiota in the rumen function has been recognized for decades; however, the dynamic changes of the ruminal microbiota and metabolic profiles in animals limit-fed high-concentrate diets remain unclear.

As cows' health, growth, and metabolism are largely dependent on metabolite production in the rumen, a comprehensive analysis of the composition of rumen fluid could offer important insights into the role of rumen-diet interactions on integrated cows' conditions (Saleem et al., 2013). About 55–60% of rumen fluid metabolites were related to the rumen microbiota (Saleem et al., 2013). Henderson et al. (2015) established a varying rumen microbiome across a wide range of host species and dietary conditions worldwide, showing a more prominent effect of the diet than of the host. However, there is a gap between the information on the rumen microbiome and that on microbial metabolism that needs to be bridged (Bannink et al., 2016). Metabolomics has become an emerging research area that quantitatively measures small molecular metabolites in biological samples (biofluids or tissues) using high-throughput approaches, such as nuclear magnetic resonance (NMR) (Zhao et al., 2014), liquid chromatography tandem–mass spectrometry (LC-MS) (Tian et al., 2016), and gas chromatography–mass spectrometry (GC-MS) (Hua et al., 2017). Gas chromatography time-of-flight/mass spectrometry (GC-TOF/MS) has been widely used in ruminant biofluid detection (Saleem et al., 2013; Sun et al., 2015; Mao et al., 2016) because of its high sensitivity in metabolite analysis. The identification and integrative analysis of these metabolites can enable a comprehensive characterization of metabolism mechanisms at the molecular level.

Nowadays, the more recently developed high-throughput MiSeq sequencing technique has been successfully used to reveal the changes and functions of the ruminal bacteria community (Mao et al., 2015; Jin et al., 2016; Ye et al., 2016). An integrated investigation of the compositions and abundances of ruminal bacteria, ciliates, fungi, and archaea could give a better understanding of the microbiota in heifers limit-fed with high-concentrate diets. We hypothesized that a wide range of dietary F:C would affect the ruminal microbiota and metabolites. The objective of this study, using four dietary levels of F:C, was to evaluate both linear and non-linear effects of increasing the proportion of dietary concentrate on ruminal profiles of microbiota (bacteria, protozoa, anaerobic fungi, and archaea) and metabolites in limit-fed Holstein heifers through a combination of the MiSeq sequencing strategy and the GC-TOF/MS technique. Moreover, the relationships between ruminal microbiota abundance and metabolites were also analyzed in the present study.

## Materials and methods

### Animals, diets, and experimental design

Twenty-four half-sib Holstein heifers (8–10 months and 263 ± 30 kg of body weight) with similar body condition from the Sanyuan Dairy Group (Beijing, China) were randomly assigned into four groups and fed diets with four different F:C (80:20, 60:40, 40:60, and 20:80; namely groups C20, C40, C60, and C80, respectively) with corn silage as the sole forage (Table S1). Half-sib heifers were used here to minimize the difference of individual animals. All heifers were fed with an adaptation diet containing 50% forage and 50% concentrate (Table S1) for 4 weeks, and then transferred to treatment diets that were formulated to meet the requirements of average daily gain at 800 g for dairy cattle (NRC, 2001). The amount of feed offered was adjusted weekly based on body weight, and the quantity of diets provided to high-concentrate-fed groups was restricted so that the intake of metabolizable energy (ME) was similar among groups. Heifers were individually fed with total mixed ration twice daily at 12 h intervals (0700 and 1900 h). Dry matter intake (DMI) for each individual heifer was recorded daily. Additionally, diets were formulated to maintain similar crude protein (CP):ME ratios in order to minimize potential effects due to differences in CP intake across diet treatments. Water was available *ad libitum*. Heifers were housed in individual stalls in a ventilated, environmentally controlled tie-stall barn with rubber mattress bedding, and were allowed access to an exercise lot for 2 h on days when sampling was not occurring.

This study was carried out in accordance with the recommendations of Instructive Notions with Respect to Caring for Experimental Animals, Ministry of Science and Technology of China. The protocol was approved by the Ethical Committee of the College of Animal Science and Technology of China Agricultural University.

### Rumen sample collection and measurements

At the end of the feeding period (day 28), rumen content (liquid and small particles; 100 mL/heifer) was collected using an oral stomach tube, approximately 4 h after morning feeding, according to a previously reported procedure (Shen et al., 2012). The device was thoroughly cleaned using fresh warm water between sample collections, and the first fraction of the sample from each cow was always discarded to ensure that sample collected was identified for each individual cow without contaminants from previous animals and her own saliva. Two mL of each rumen sample was immediately frozen in liquid nitrogen and stored at −80°C to minimize any possible microbial activities for use in later DNA extraction. Another 2 mL of each rumen sample was passed through a disposable 0.22 μm filter, and after freezing in liquid nitrogen, samples were stored at −80°C for future ruminal metabolomic analysis. Rumen pH was immediately measured after collection by a mobile pH meter (Starter 300; Ohaus Instruments Co. Ltd., Shanghai, China). After pH measurement, samples were passed through four layers of sterile cheesecloth and kept on ice until further processing. Filtered rumen fluid was centrifuged (17,000 g for 30 min at 4°C) to obtain a clear supernatant, which was further analyzed for NH_3_-N using a phenol-hypochlorite assay (Broderick and Kang, 1980). Freshly prepared metaphosphoric acid (25% w/v; 2 mL) was added to 8 mL of filtered rumen fluid, vortexed, and then centrifuged (17,000 g for 10 min at 4°C). The supernatant was stored at −20°C for further measurement of VFAs. The VFA concentrations were determined with a capillary column gas chromatograph (Cao et al., 2008).

### Microbial DNA extraction, PCR amplification, illumina miseq sequencing, and sequencing data processing

Genomic DNA was extracted from the rumen samples using a PowerSoil DNA Isolation Kit (Mobio, Carlsbad, CA, USA), following the manufacturer's protocol. The extracted DNA was subjected to PCR amplification in triplicate using the Accuprime Taq DNA Polymerase System (Invitrogen, Carlsbad, CA, USA). For bacterial analysis, the V3-V4 region of the 16S rRNA gene was amplified using primers 338F (5′-ACTCCTRCGGGAGGCAGCAG-3′) and 806R (5′-GGACTACCVGGGTATCTAAT-3′) (Mao et al., 2015). For ciliate protozoal analysis, the V3-V4 and signature regions 1–2 of the 18S rRNA gene were amplified using primers P-SSU-316F (5′-GCTTTCGWTGGTAGTGTATT-3′) (Sylvester et al., 2004) and GIC758R (5′-CAACTGTCTCTATKAAYCG-3′) (Ishaq and Wright, 2014). For fungal analysis, the internal transcribed spacer region 1 (ITS1) of the fungal rRNA gene was amplified using primers ITS1-F (5′-CTTGGTCATTTAGAGGAAGTAA-3′) (Hristov et al., 2013) and ITS1-R (5′-GCTGCGTTCTTCATCGATGC-3′) (Kumar et al., 2015). For archaeal analysis, the V3-V4 region of the 16S rRNA gene was amplified using primers F341 (5′-CTACGGGGYGCASCAG-3′) (Wei et al., 2013) and R806 (5′-GGACTACVVGGGTATCTAATC-3′). Barcodes of an eight-base sequence unique to each sample were added to each primer for sample identification. Amplification was performed as follows: an initial denaturing step of 95°C for 3 min; followed by 25 cycles of denaturing (95°C for 30 s), annealing (60°C for bacteria/54°C for protozoa/50°C for fungi/55°C for archaea, for 20 s), and elongation (72°C for 40 s); and a final extension step of 72°C for 10 min. Amplicons were extracted from 2% agarose gels and purified using an AxyPrep DNA Gel Extraction Kit (Axygen Bioscience, Union City, CA, USA), following the manufacturer's instructions, and quantified using QuantiFluor-ST (Promega, USA). Illumina TruSeq PE Cluster and a SBS Kit were used to perform cluster generation, template hybridization, isothermal amplification, linearization, blocking, denaturation, and hybridization of the sequencing primers, according to the manufacturer's instructions. Paired-end sequencing, 2 × 250 bp, was performed to sequence all libraries on an Illumina MiSeq platform according to standard protocols (Caporaso et al., 2012).

The sequences obtained from the MiSeq platform were processed through open-source software pipeline QIIME (Quantitative Insights into Microbial Ecology) version 1.8.0-dev (Caporaso et al., 2010b), with the criteria as described by previous reports (Mao et al., 2015; Ye et al., 2016). Briefly, (1) the reads that had a mean quality score of no <20 and no shorter than 50 bp were retained; (2) discarding occurred for reads that had exact barcode matching, two nucleotide mismatches in primer matching, and ambiguous characters; (3) only sequences that overlapped by more than 10 bp were assembled according to their overlap sequence. Reads that could not be assembled were discarded. Sequences were binned into operational taxonomic units (OTUs) based on 97% identity using UCLUST (version 7.1, http://drive5.com/uparse/), and chimeric sequences were identified and removed by UCHIME (Edgar, 2010). The most abundant sequence within each OTU from specific libraries (bacteria, protozoa, fungi, and archaea) was designated as the “representative sequence” and aligned against the SILVA bacterial database (version 119) (Pruesse et al., 2007), NCBI-nt protozoa database (Koetschan et al., 2014), Unite fungi ITS database (version 7.0) (Koljalg et al., 2005), and SILVA archaea database using PYNAST (Caporaso et al., 2010a), respectively, with the default parameters set by QIIME. Community richness and diversity, analyzed with measures, such as Good's coverages, observed species, PD whole tree, Pielou, Chao1 and Shannon indices, weighted uniFrac distance-based principal coordinate analysis (PCoA), and weighted distance-based analysis of molecular variance (AMOVA), which were used to illustrate significant differences among the samples, were assessed by the program MOTHUR v.1.35.0 (Schloss et al., 2009).

All the raw sequences (16S, 18S, and ITS) after assembling and filtering were submitted to the NCBI Sequence Read Archive (SRA; http://www.ncbi.nlm.nih.gov/Traces/sra/), under accession number SRP089832.

### Preparation of rumen sample for GC-TOF/MS, and identification of compounds discovered

The samples of rumen fluid were prepared, detected, and identified using the procedure previously described by Sun et al. (2015), with modifications. Adonitol was used as an internal standard and GC-TOF/MS analysis was performed using an Agilent 7890 gas chromatograph system coupled with a Pegasus HT TOF/MS (LECO, St. Joseph, MI, USA). The system had a DB-5MS capillary column (30 m × 250 μm inner diameter, 0.25 μm film thickness; J&W Scientific, Folsom, CA, USA) coated with 5% diphenyl cross-linked with 95% dimethylpolysiloxane. The temperature was initially kept at 50°C for 1 min, and then increased to 320°C at a rate of 10°C/min; the column was then maintained for 5 min. The temperatures of injection, transfer line, and ion source were 280, 270, and 220°C, respectively. The mass spectrometry data were acquired in full-scan mode with a mass-to-change ratio (*m*/*z*) range of 30–600 at a rate of 20 spectra/s after a solvent delay of 366 s.

The LECO-Fiehn Rtx5 database (Kind et al., 2009) and commercial databases, including KEGG (http://www.genome.jp/kegg/) and NIST (http://www.nist.gov/index.html), were utilized to further identify and validate different metabolites. The resulted data involving the peak number, sample name, and normalized peak area were fed into the SIMCA software package (V14, Umetrics AB, Umea, Sweden) for principal component analysis (PCA), and orthogonal projections to latent structures-discriminant analysis (OPLS-DA). OPLS-DA was used here to obtain maximal covariance between measured data and response variable. The parameters of variable importance projection (VIP) value >1.5, and *q* (false discovery rate, FDR) < 0.05, were used as criteria to identify different metabolites between every two dietary treatment groups. In order to find the varying patterns of different metabolites with increasing dietary concentrate levels, the linear, quadratic, and cubic effects among treatments were evaluated among all different metabolites from six comparisons.

### Correlations between microbial communities and rumen metabolites

Rumen metabolites with VIP > 1.5, *q* < 0.05, similarity > 600 and fold change > 2 or < 0.5, main VFAs, and microbe genera that were significantly affected by dietary treatments (*q* < 0.05 and relative abundance >0.5% in at least one of the samples) were used for interactive analysis in R (V3.2.4, http://www.r-project.org/). Spearman's rank correlations and *q*-values were calculated using the Psych packages (http://cran.r-project.org/web/packages/psych; author, W. Revelle; published date, 2016; version, 1.6.9; FDR correct was embedded in the package). Correlations had an absolute Spearman's correlation of no <0.55, with a FDR level under 0.05. These correlations were visualized using the Pheatmap (https://cran.r-project.org/package=pheatmap; author, R. Kolde; published date, 2015; version, 1.0.8) package in R and Cytoscape 2.8.2 (Smoot et al., 2011).

### Data analysis

Statistical analysis was performed using R software packages. Data of microbial abundance were transformed to log_10_ (*n* + 1) if necessary to ensure normal distribution (Jin et al., 2016). The dietary effects were tested using the Estimability package (https://cran.r-project.org/package=estimability; author, R. V. Lenth; published date, 2015; version, 1.1-1) in R. The lm function of the Estimability package in R was used to evaluate the linear, quadratic, and cubic effects of the dietary concentrate levels on the variables. Standard errors of the mean were reported. Significance was declared at *P* < 0.05. Also, all *P* values from the multiple comparison analyses of the ruminal microbial community and the metabolomics data were adjusted by the FDR by the BonEV package (https://cran.r-project.org/package=BonEV; author, D. Li; published date, 2016; version, 1.0) with *p. adjust* in R. FDR-corrected *P* values below 0.05 (*q* < 0.05) were considered significantly different.

## Results

### Intake and rumen fermentation parameters

DMI (5.32, 4.97, 4.69, and 4.42 kg/day) quadratically decreased (*P* = 0.02) with increasing dietary concentrate levels. As the level of dietary concentrate increased, rumen pH, ratio of acetate:propionate, and the proportion of acetate linearly decreased (*P* < 0.01), while the proportions of propionate and butyrate linearly increased (*P* < 0.01), the concentration of NH_3_-N quadratically increased (*P* < 0.01) with C80 at the highest value and C40 at the lowest value, the proportion of isobutyrate quadratically increased (*P* = 0.02) with C40 at the highest value and C80 at the lowest value, and the proportion of isovalerate cubically changed (*P* = 0.04) with C60 at the highest value and C40 at the lowest value (Table 1).

**Table 1:** Rumen fermentation parameters affected by increasing levels of concentrate in diets.

**Items**	**Treatments^a^**				**SEM^b^**	***P*****-value**			
	**C20**	**C40**	**C60**	**C80**		**Treatment**	**Linear**	**Quadratic**	**Cubic**
pH	7.02	6.86	6.80	6.59	0.044	<0.01	<0.01	0.60	0.37
NH_3_-N, mg/dL	2.40	2.22	3.93	8.27	0.583	<0.01	<0.01	<0.01	0.78
TVFA, mM	86.10	89.53	92.49	94.64	1.806	0.43	0.11	0.87	0.98
**VFAs, molar % of TVFA**									
Acetate	65.97	62.48	58.04	53.85	1.135	<0.01	<0.01	0.81	0.85
Propionate	20.99	22.10	23.31	25.44	0.624	0.04	<0.01	0.63	0.86
Butyrate	9.69	11.05	12.42	16.91	0.688	<0.01	<0.01	0.09	0.43
Valerate	0.52	0.58	1.08	0.56	0.100	0.11	0.42	0.11	0.08
Isobutyrate	1.24	2.29	2.16	0.93	0.226	0.10	0.59	0.02	0.97
Isovalerate	1.59	1.49	2.99	2.27	0.223	0.05	0.06	0.43	0.04
Acetate: Propionate	3.18	2.89	2.53	2.16	0.115	<0.01	<0.01	0.81	0.96

a *C20, diet contained 20% of concentrate; C40, diet contained 40% of concentrate; C60, diet contained 60% of concentrate; C80, diet contained 80% of concentrate*.

b *SEM, standard error of the mean*.

### Ruminal bacteria changes within the four treatment groups

**Figure 1:**
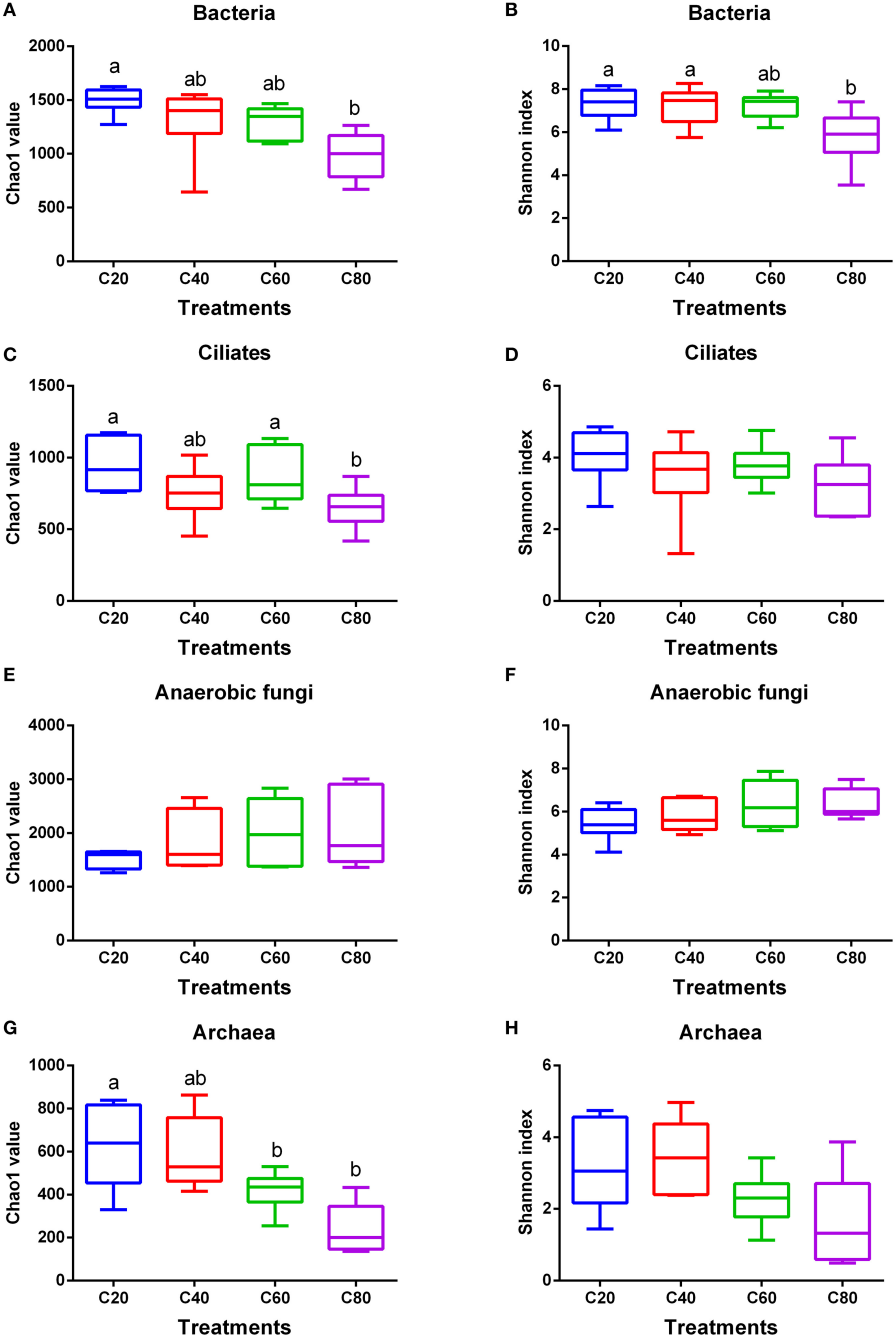
Changes in ruminal microbial richness and diversity with increasing levels of concentrate in diets. Bacterial **(A)**, ciliate protozoal **(C)**, anaerobic fungal **(E)**, and archaeal **(G)** richness estimated by the Chao1 value. Bacterial **(B)**, ciliate protozoal **(D)**, anaerobic fungal **(F)**, and archaeal **(H)** diversity estimated by Shannon index. Boxes represent the interquartile range (IQR) between the first and third quartiles (25 and 75th percentiles, respectively), and the horizontal line inside the box defines the median. Whiskers represent the lowest and highest values within 1.5 times the IQR from the first and third quartiles, respectively. Boxes with the different letters above their whiskers are significantly different (*P* < 0.05) among treatments. C20, diet containing 20% of concentrate; C40, diet containing 40% of concentrate; C60, diet containing 60% of concentrate; C80, diet containing 80% of concentrate.

In total, 1,888,443 raw reads were obtained for bacterial 16S rRNA genes by the sequencing procedure. After screening with strict criteria (as described in the methods), 1,797,207 valid reads were obtained, accounting for 95.2% of the raw reads. The Good's coverages for all samples were more than 99%. Compared with other groups, the C80 group had the lowest observed species, PD whole tree, and Pielou (*P* < 0.05; Table 2). Similar to C60 and C40 groups, the C80 group supported significantly less richness (*P* < 0.05) than that of the C20 group based on Chao1 index (Figure 1A). Shannon index analysis indicated a similar tendency of diversity (Figure 1B).

**Table 2:** Alpha diversity index of ruminal bacteria, protozoa, fungi, and archaea among all treatments.

**Microbiota**	**Indices**	**Treatments^a^**				**SEM^b^**	***P*-value**
		**C20**	**C40**	**C60**	**C80**		
Bacteria	Observed species	1245^a^	1104^a^	1039^ab^	774^b^	80.1	0.01
	PD whole tree	106^a^	99^ab^	96^ab^	79^b^	4.9	0.01
	Pielou	0.71^a^	0.71^a^	0.71^a^	0.59^b^	0.029	0.03
Protozoa	Observed species	523	410	438	347	41.7	0.05
	PD whole tree	4	3	4	3	0.4	0.09
	Pielou	0.42	0.38	0.41	0.36	0.031	0.56
Fungi	Observed species	955	1,117	1,263	1,204	133.2	0.47
	PD whole tree	191	277	259	215	40.5	0.48
	Pielou	0.53	0.56	0.60	0.61	0.025	0.15
Archaea	Observed species	525^a^	503^a^	329^ab^	187^b^	55.7	<0.01
	PD whole tree	40^a^	41^a^	31^ab^	21^b^	3.4	0.02
	Pielou	0.35	0.38	0.27	0.22	0.047	0.09

ab *Different letters within a row means values with significantly different (P < 0.05)*.

a *C20, diet contained 20% of concentrate; C40, diet contained 40% of concentrate; C60, diet contained 60% of concentrate; C80, diet contained 80% of concentrate*.

b *SEM, standard error of the mean*.

**Figure 2:**
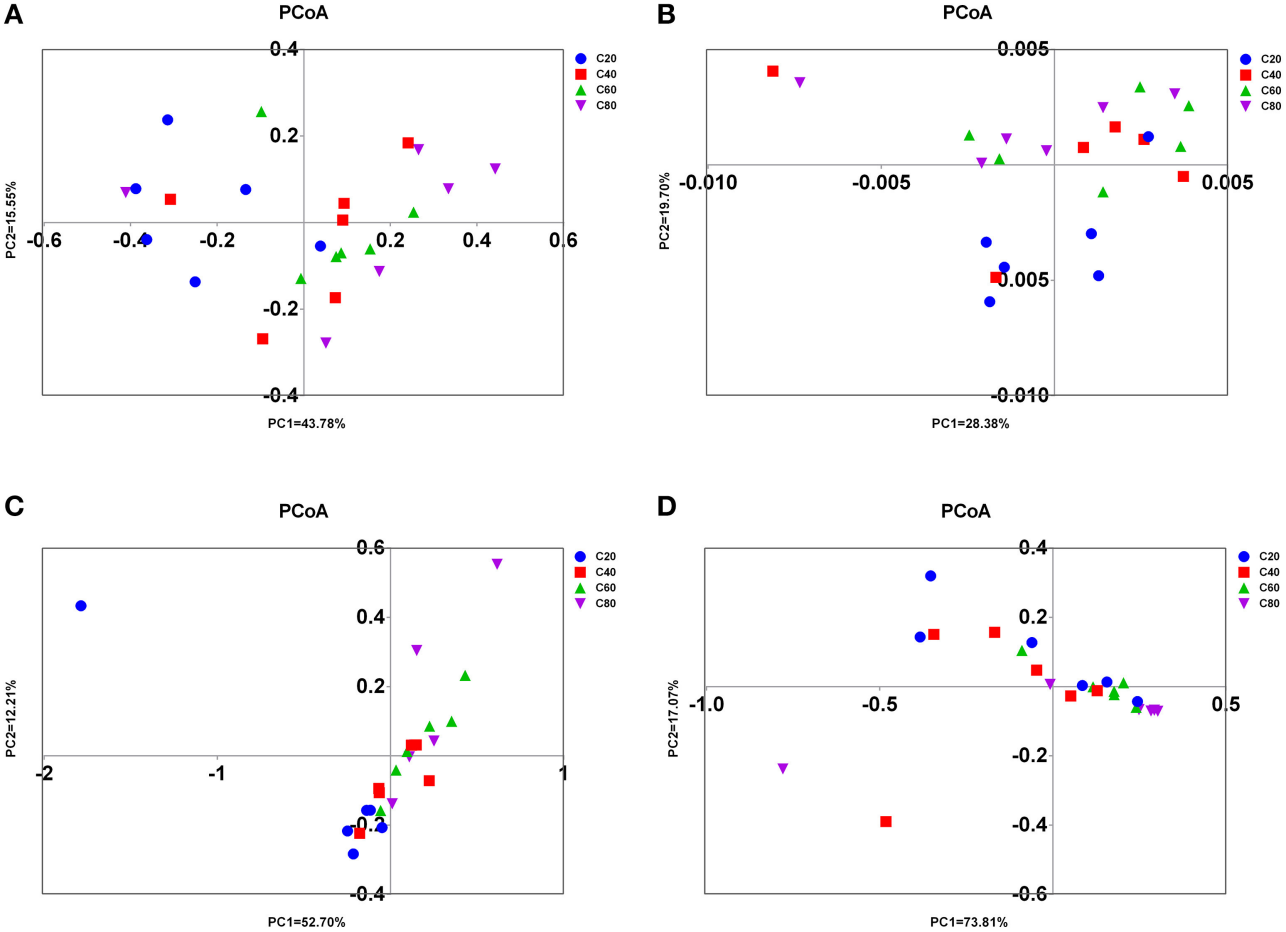
PCoA of rumen microbial communities. Weighted PCoA by ruminal bacteria **(A)**, ciliate protozoa **(B)**, anaerobic fungi **(C)**, and archaea **(D)** microbiota. C20, diet containing 20% of concentrate; C40, diet containing 40% of concentrate; C60, diet containing 60% of concentrate; C80, diet containing 80% of concentrate.

Significant differences were found in the microbial communities at the OTU level between C80 and C40 groups (AMOVA, *P* = 0.03), between C80 and C20 groups (*P* < 0.01), between C60 and C20 groups (*P* < 0.01), and between C40 and C20 groups (*P* = 0.04). There was no significant difference found in the microbial compositions between C80 and C60 groups (*P* = 0.12), and between C60 and C40 groups (*P* = 0.46). The results of PCoA with weighted uniFrac distances indicated that the four treatment groups were largely separated from each other at the OTU level (Figure 2A). There were 2,095 OTUs identified in total, among which 1,352 OTUs were found in all four treatment groups and accounted for 64.5% of the total OTUs, revealing the presence of a strong common microbiota (Figure S1A). OTU2, which was classified into the family *Succinivibrionaceae*, was the most dominant OTU taking up 6.73% of the total OTUs.

There were 24 bacterial phyla identified in the rumen samples. Among these phyla, *Bacteroidetes, Firmicutes*, and *Proteobacteria* had relatively high abundance, at mean abundance levels of 57.2 ± 2.6% (mean ± standard error of the mean), 26.8 ± 1.6%, and 10.3 ± 2.2%, respectively (Figure S2A). With increasing dietary concentrate levels, the relative abundance of *Fibrobacteres* (*q* < 0.01) and SR1 (*q* = 0.04) linearly decreased.

**Figure 3:**
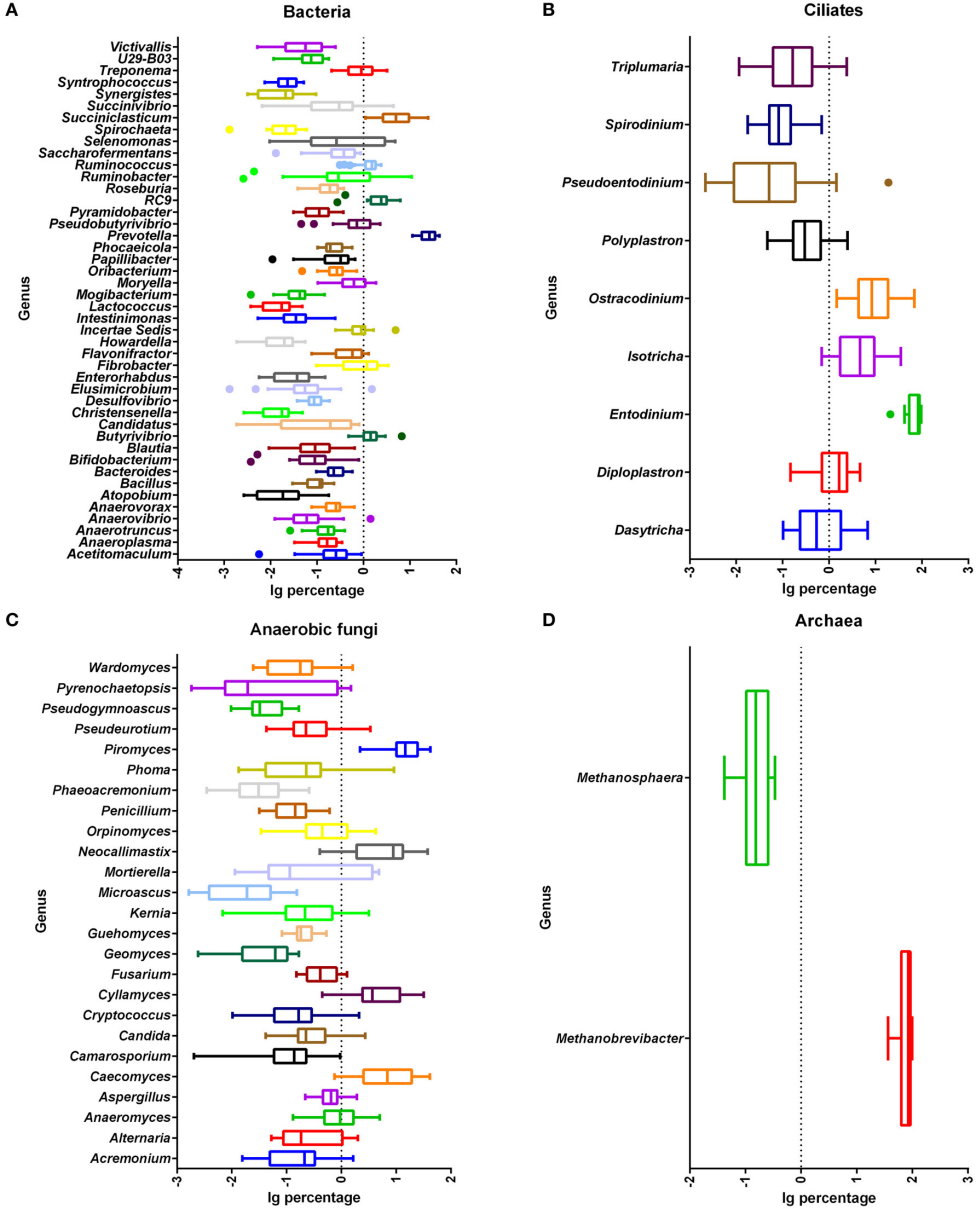
Relative abundance of shared microbial genera across all rumen samples. Abundance of genera of bacteria **(A)**, ciliate protozoa **(B)**, anaerobic fungi **(C)**, and archaea **(D)**. Percentage after log transformation is shown on the *x*-axis. Boxes represent the interquartile range (IQR) between the first and third quartiles (25 and 75th percentiles, respectively), and the line inside the box defines the median. Whiskers represent the lowest and highest values within 1.5 times the IQR from the first and third quartiles, respectively. Samples with a relative abundance for a given genus exceeding those values are represented as points beside the boxes.

There were 140 bacterial taxa identified at the genus level through analysis of microbiota compositions, and 44 genera were present in all samples, which was indicative of the core microbiome in this study (Figure 3A). Among these, *Prevotella* (22.6 ± 1.9%), *Succiniclasticum* (7.0 ± 1.3%), RFN43 (3.0 ± 0.5%), RC9 (2.4 ± 0.3%), *Butyrivibrio* (1.7 ± 0.3%), *Ruminococcus* (1.4 ± 0.1%), *Ruminobacter* (1.4 ± 0.5%), *Fibrobacter* (1.2 ± 0.2%), and *Selenomonas* (1.2 ± 0.3%) were considered as high abundance taxa (Figure S3A). As the proportion of concentrate increased in diets, the percentage of *Enterorhabdus* (*q* < 0.01), *Blautia* (*q* = 0.03), *Anaerosporobacter* (*q* = 0.02), *Fibrobacter* (*q* < 0.01), *Succinimonas* (*q* < 0.01), RFN54 (*q* < 0.01), *Victivallis* (*q* = 0.03), *Bilophila* (*q* = 0.03), *Saccharofermentans* (*q* = 0.02), and *Anaeroplasma* (*q* = 0.03) linearly decreased, and that of *Acidithiobacillus* (*q* = 0.01) quadratically decreased (Table 3). On the contrary, the proportions of *Christensenella* (*q* = 0.02) and *Turicibacter* (*q* = 0.03) linearly increased with the increasing level of concentrate in diets.

**Table 3:** Main microbiota (accounting for ≥0.05% of the total sequences in at least one of the samples) that significantly changed among different treatments (abundance of the genera is expressed as a percentage).

**Items**	**Genera level**	**Treatments^a^**				**SEM^b^**	***q*****-value^c^**			
		**C20**	**C40**	**C60**	**C80**		**Treatment**	**Linear**	**Quadratic**	**Cubic**
Bacteria	*Enterorhabdus*	0.09	0.06	0.03	0.01	0.010	0.01	<0.01	0.68	1.00
	*Blautia*	0.26	0.16	0.08	0.04	0.037	0.13	0.03	0.68	1.00
	*Anaerosporobacter*	0.48	0.08	0.05	0.06	0.047	0.01	0.02	0.31	0.84
	*Fibrobacter*	2.35	1.52	0.76	0.36	0.211	<0.01	<0.01	0.63	1.00
	*Bacillus*	0.12	0.10	0.14	0.05	0.015	0.05	0.16	0.39	0.41
	*Succinimonas*	0.43	0.08	0.04	0.04	0.038	<0.01	0.00	0.17	0.72
	*Victivallis*	0.17	0.06	0.04	0.04	0.021	0.03	0.03	0.39	0.95
	*Schwartzia*	0.01	0.04	0.06	0.23	0.032	0.12	0.06	0.51	0.96
	*Saccharofermentans*	0.58	0.56	0.33	0.17	0.080	0.07	0.02	0.63	0.95
	*Anaeroplasma*	0.25	0.22	0.17	0.09	0.030	0.08	0.03	0.68	1.00
Protozoa	*Polyplastron*	1.14	0.27	0.35	0.21	0.137	0.07	0.04	0.57	0.83
	*Ostracodinium*	32.12	14.24	8.63	5.00	4.332	0.10	0.04	0.57	0.83
	*Entodinium*	50.66	74.40	76.95	87.19	6.643	0.10	0.04	0.61	0.83

a *C20, diet contained 20% of concentrate; C40, diet contained 40% of concentrate; C60, diet contained 60% of concentrate; C80, diet contained 80% of concentrate*.

b *SEM, standard error of the mean*.

c *q-value, false discovery rate; significance were considered at q < 0.05*.

### Ruminal ciliate protozoa changes within the four treatment groups

A total of 1,311,850 sequences were derived from the ciliate protozoa 18S rRNA genes by sequencing, with 1,311,783 used for analysis after quality control at 54,658 sequences per sample on average. The Good's coverages for all samples were more than 99%. There were no significant differences found in observed species, PD whole tree, and Pielou among treatments (Table 2). The Chao1 value was indicative that the C80 group had lower (*P* < 0.05) richness than that of the C60 and C20 groups, but the richness in the C40 group was similar with that of other three groups (Figure 1C). However, the Shannon index values were suggestive that ciliate community diversities were similar among the four groups (Figure 1D).

Significant differences were found in the ciliate communities at the OTU level between C80 and C40 groups (AMOVA, *P* = 0.02), between C80 and C20 groups (*P* < 0.01), and between C60 and C20 groups (*P* < 0.01). There were no significant differences found in the ciliate compositions between C80 and C60 groups (*P* = 0.10), between C60 and C40 groups (*P* = 0.11), and between C40 and C20 groups (*P* = 0.14). The PCoA with weighted uniFrac distances also showed that C80 was separated from the C40 and C20 groups at the OTU level (Figure 2B). There were 2,782 OTUs identified in total, and 641 OTUs were found in all four treatment groups (23.04%), which represented the common ciliates (Figure S1B).

The ciliate sequences detected at the phylum level largely belonged to Alveolata (>99.9%). There were 34 taxa identified at the genus level through analysis of microbiota composition, and nine genera were present in all samples, which were indicative of the core protozoal community in this study (Figure 3B). *Entodinium* were the most abundant group among ciliates detected at the genus level, with a mean relative abundance of 72.3 ± 4.3%, followed by the second most abundant genus group of *Ostracodinium* (15.0 ± 3.2%) (Figure S3B). With an increasing amount of concentrate in diets, the prevalence of *Entodinium* linearly increased (*q* = 0.04), while the relative abundance of *Ostracodinium* (*q* = 0.04) and *Polyplastron* (*q* = 0.04) linearly decreased (Table 3).

### Ruminal anaerobic fungal changes within the four treatment groups

In total, 1,447,310 valid sequences were obtained for the anaerobic fungal ITS1 genes by sequencing, and 1,231,562 valid reads were obtained after screening, accounting for 85.1% of the raw reads. The Good's coverages for all samples were more than 98%. There were no significant differences found in observed species, PD whole tree, and Pielou among treatments (Table 2). An increasing amount of dietary concentrate (*P* > 0.05) did not affect the diversity and richness of the anaerobic fungal communities, shown by the Chao1 and Shannon indices (Figures 1E,F).

There were significant differences found in the microbial communities at the OTU level between C80 and C20 groups (AMOVA, *P* = 0.02), and between C60 and C20 groups (*P* < 0.05). The PCoA axis 1 accounted for 52.70% of the variation, and the PCoA axis 2 accounted for 12.21% of the variation (Figure 2C). In total, 7,047 OTUs were identified, and 1,441 OTUs were found in all four treatment groups (20.45%), which represented the common anaerobic fungi in the rumen (Figure S1C).

Ruminal anaerobic fungi detected from the present study represented more than nine separate phyla (Figure S2B). The predominant anaerobic fungi detected were Neocallimastigomycota, Ascomycota, Unclassified Fungi, Zygomycota, and Basidiomycota, with the mean relative abundance of 64.3 ± 3.8%, 22.6 ± 2.4%, 10.0 ± 0.4%, 1.4 ± 0.4%, and 1.3 ± 0.2%, respectively. As the proportion of dietary concentrate increased, the relative abundance of Ascomycota (*q* < 0.01), Basidiomycota (*q* = 0.03), Cercozoa (*q* < 0.01), and Chytridiomycota (*q* = 0.04) linearly increased; meanwhile, the relative abundance of Neocallimastigomycota linearly decreased (*q* = 0.03).

There were 335 genera identified at the genus level, and 25 genera were present in all samples, which were indicative of the core fungi community in this study (Figure 3C). *Piromyces, Caecomyces, Neocallimastix, Cyllamyces*, and *Anaeromyces* were the predominant genera, with mean relative abundances of 17.9 ± 2.3%, 11.1 ± 2.1%, 9.3 ± 1.7%, 8.5 ± 1.8%, and 1.4 ± 0.3%, respectively (Figure S3C). However, no genus was significantly affected by dietary concentrate levels (*q* > 0.10).

### Ruminal archaea changes within the four treatment groups

After quality control, a total of 873,225 clean reads were derived from the archaea 16S rRNA genes by sequencing, with a mean length of 391 bp. The Good's coverages for all samples were more than 99%. Compared with other groups, the C80 group had the lowest observed species and PD whole tree (*P* < 0.05; Table 2). The C80 and C60 groups supported significantly lower richness (*P* < 0.05) than that of the C20 group, based on Chao1 index (Figure 1G). No significant difference of archaea community diversities was found among treatments, based on the Shannon index (Figure 1H).

A significant difference was only found in the microbial communities at the OTU level between C60 and C40 groups (AMOVA, *P* < 0.05). The results of PCoA with weighted uniFrac distances was indicative that the four treatment groups have a better separation at PCoA axis 1 than at PCoA axis 2 (73.81 vs. 17.07%, Figure 2D). In total, 1,627 OTUs were identified, and 331 OTUs were found in all four treatment groups (20.34%) which represented the common anaerobic fungi in the rumen (Figure S1D).

The sequences detected at the phylum level largely belonged to *Euryarchaeota* (>86.3%), with only a few sequences belonging to *Thaumarchaeota* as the remaining phylum. Unclassified sequences comprised 13.65% of the total reads. There were seven taxa identified at the genus level through analysis of microbiota composition, and two genera were present in all samples, which were indicative of the core archaeal community in this study (Figure 3D). The most abundant known genera was *Methanobrevibacter* (78.0 ± 3.7%) (Figure S3D). Other minor genera, such as *Methanosarcina, Methanosphaera*, and *Methanimicrococcus*, accounted for 0.21 ± 0.15%, 0.18 ± 0.02%, and 0.05 ± 0.02%, respectively, with several reads belonging to *Nitrososphaera, Methanocorpusculum*, and *Halostagnicola*. The remainder of the sequences (21.60%) was unclassified at the genus level. However, no genus was significantly affected by dietary concentrate levels (*q* > 0.10).

### Identification, quantification, and statistical comparison of GC-TOF/MS metabolites in the rumen

As shown in the GC-TOF/MS total ion chromatograms of rumen samples from heifers fed diets with increasing levels of concentrate, there were in total 558 valid peaks identified from the four groups (Figure S4). Based on the LECO/Fiehn Metabolomics Library, 243 metabolites, including amino acids, fatty acids, sugars, and organic acids, were quantified in the rumen from the four groups (Table S2), and the four groups shared the same metabolite categories.

**Figure 4:**
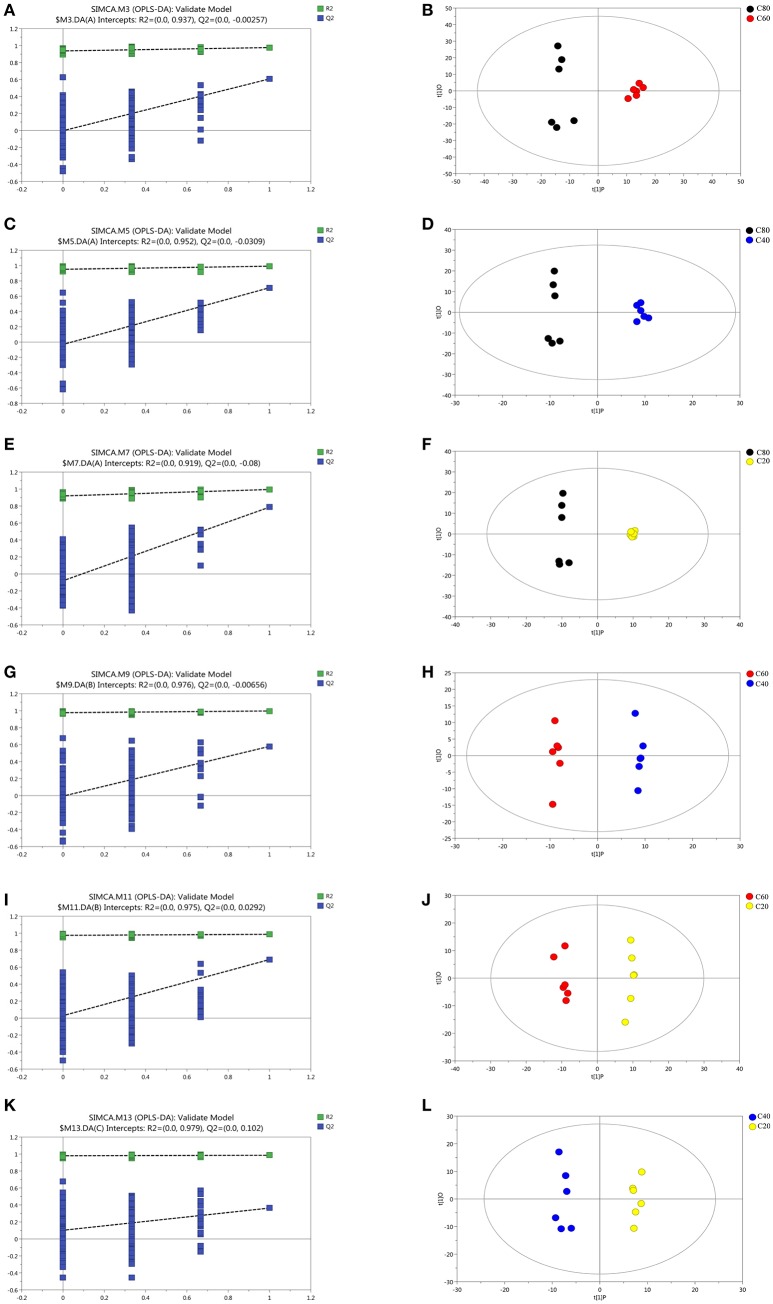
Corresponding validation plots and OPLS-DA score plots derived from the GC-TOF/MS metabolite profiles of rumen samples for cows fed increasing levels of concentrate in their diets. Corresponding validation plots and OPLS-DA score plots (respectively) for: **(A,B)** the C80 group vs. C60 group; **(C,D)** the C80 group vs. C40 group; **(E,F)** the C80 group vs. C20 group; **(G,H)** the C60 group vs. C40 group; **(I,J)** the C60 group vs. C20 group; **(K,L)** the C40 group vs. C20 group. C20, diet containing 20% of concentrate; C40, diet containing 40% of concentrate; C60, diet containing 60% of concentrate; C80, diet containing 80% of concentrate.

Data of the GC-TOF/MS spectra among the four groups analyzed using PCA scores plots are listed in Figure S5. The parameters for the assessment of the OPLS-DA model quality in discriminating the four groups could be represented by validation plots (Figures 4A,C,E,G,I,K). The corresponding R^2^Y values of OPLS-DA models in C80 vs. C60, C80 vs. C40, C80 vs. C20, C60 vs. C40, C60 vs. C20, and C40 vs. C20 were 0.937, 0.952, 0.919, 0.976, 0.975, and 0.979, respectively. The permutation tests of the four groups were all in a better range with the *R*^2^-values of the four groups all >0.919, indicating a satisfactory effectiveness of the model, which can be used to identify the difference between two treatments. The OPLS-DA score results of the six comparisons were shown in Figures 4B,D,F,H,J,L. All the samples in the score plots were within the 95% of the Hotelling T^2^ ellipse.

### Metabolomic profiles in the rumen

In total, 16 different metabolites (VIP > 1.5 and *q* < 0.05) were obtained from the comparisons of C80 vs. C60, C80 vs. C40, C80 vs. C20, and C60 vs. C20 (Table S2 and Table 4). Among them, three metabolites were classified into amino acids, peptides and analogs (super class level; the same as below), three metabolites were classified into lipids, five metabolites were classified into aromatic homomonocyclic compounds, one metabolite was classified into organic acids and derivatives, and one metabolite was classified into carbohydrates, and three metabolites were unclassified. With the increasing of dietary concentrate levels, six metabolites (3-aminoisobutyric acid, proline, 3,4-dihydroxyphenylacetic acid, phenylacetic acid, uracil, and beta-sitosterol) linearly increased (*q* < 0.01 to *q* = 0.03), five metabolites [O-methylthreonine, bis(2-hydroxypropyl)amine, hydrocinnamic acid, 4-aminobenzoic acid, and phytanic acid] linearly decreased (*q* < 0.01 to *q* = 0.02), pyruvic acid (*q* < 0.01) quadratically decreased with the C60 group having the highest value and the C80 group having the lowest value, while 5-methylresorcinol (*q* = 0.04) and gluconic lactone (*q* = 0.04) significantly changed (Table 4).

**Table 4:** Comparisons of ruminal metabolites that significantly changed in the four treatments.

**Super class**	**Metabolite names**	**Treatments^a^**				**SEM^b^**	***q*****-value^c^**			
		**C20**	**C40**	**C60**	**C80**		**Treatment**	**Linear**	**Quadratic**	**Cubic**
Aromatic homomonocyclic compounds	3,4-dihydroxyphenylacetic acid	4.44E-06	2.30E-05	6.91E-05	1.79E-04	2.061E-05	0.0	0.01	0.45	0.93
Amino acids, peptides, and analogues	3-aminoisobutyric acid	2.15E-04	2.65E-04	5.00E-04	7.52E-04	6.300E-05	0.01	<0.01	0.62	0.93
Aromatic homomonocyclic compounds	4-aminobenzoic acid	4.51E-05	3.41E-05	7.96E-06	3.12E-06	4.920E-06	<0.01	<0.01	0.87	0.76
–	5-methylresorcinol	4.52E-05	2.90E-05	5.65E-05	1.23E-05	5.014E-06	0.01	0.05	0.20	0.04
Lipids	beta-sitosterol	5.28E-06	2.78E-06	1.16E-05	4.16E-05	3.901E-06	<0.01	<0.01	0.06	0.93
–	Bis(2-hydroxypropyl)amine	7.42E-04	3.80E-04	5.76E-04	1.07E-04	7.784E-05	0.03	0.02	0.87	0.29
Carbohydrates	Gluconic lactone	5.23E-05	5.33E-05	1.02E-09	1.10E-09	6.286E-06	<0.01	<0.01	0.99	0.04
Aromatic homomonocyclic compounds	Hydrocinnamic acid	2.32E-01	1.95E-01	1.29E-01	1.14E-01	1.295E-02	<0.01	<0.01	0.76	0.76
Lipids	Linoleic acid	8.97E-04	1.40E-03	1.69E-03	7.94E-04	1.547E-04	0.15	0.99	0.12	0.81
–	O-methylthreonine	3.61E-04	2.91E-04	2.88E-04	6.56E-05	2.901E-05	<0.01	<0.01	0.19	0.45
Aromatic homomonocyclic compounds	Phenylacetic acid	4.17E-03	8.60E-03	1.80E-02	2.23E-02	2.482E-03	0.04	0.01	0.99	0.93
Lipids	Phytanic acid	1.03E-03	8.03E-04	4.69E-04	2.63E-04	8.011E-05	<0.01	<0.01	0.99	0.93
Amino acids, peptides, and analogues	Proline	2.45E-04	1.73E-04	8.03E-04	8.16E-04	1.074E-04	0.05	0.03	0.97	0.60
Organic acids and derivatives	Pyruvic acid	6.00E-05	8.48E-05	8.51E-05	4.49E-06	7.803E-06	<0.01	<0.01	<0.01	0.60
Aromatic homomonocyclic compounds	Uracil	8.94E-04	1.38E-03	2.68E-03	3.83E-03	4.181E-04	0.06	0.02	0.87	0.93
Amino acids, peptides, and analogues	Alanine	6.42E-04	9.68E-04	2.15E-03	9.14E-04	1.941E-04	0.03	0.25	0.15	0.27

a *C20, diet contained 20% of concentrate; C40, diet contained 40% of concentrate; C60, diet contained 60% of concentrate; C80, diet contained 80% of concentrate*.

b *SEM, standard error of the mean*.

c *q-value, false discovery rate; significance were considered at q < 0.05*.

### Correlations between the ruminal metabolome and microbiome

**Figure 5:**
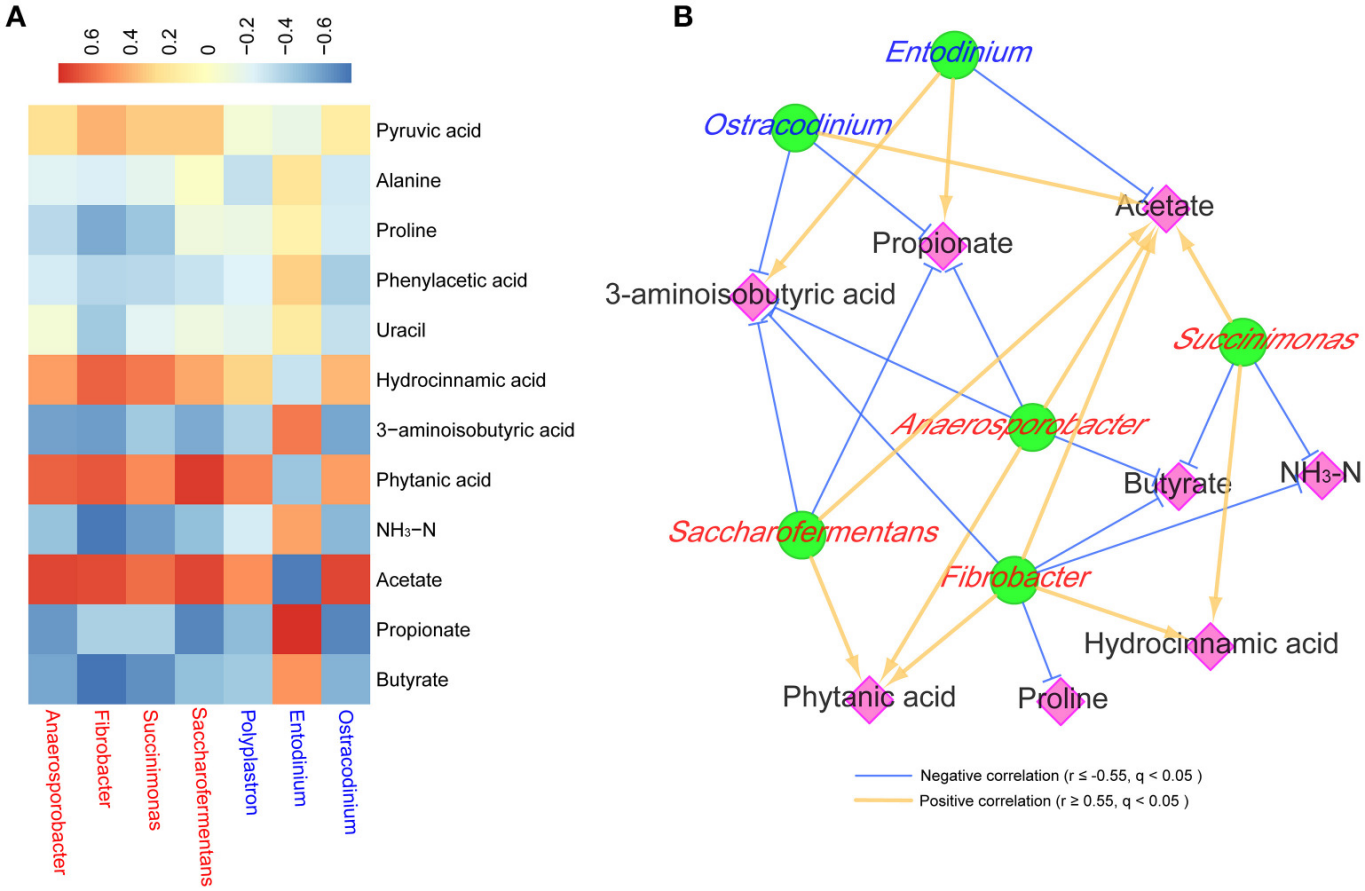
Correlations between the predominant ruminal microbiota affected by treatments and the potential marker compounds. Only the significantly affected ruminal microbiota (at the genera level) that had a mean relative abundance >0.5% were considered in the correlation. Only the ruminal metabolites that had VIP > 1.5, *q* < 0.05, similarity > 600, and fold change >2 or <0.5 were considered in the correlation. Bacteria are shown by red text color, and protozoa are shown by blue text color. **(A)** Correlation between the microbiota and metabolites. Cells are colored based on Spearman's correlation coefficient: red represents a positive correlation, and blue represents a negative correlation. **(B)** Co-occurrence network analysis among the microbiota and metabolites. Each co-occurring pair among microbial populations at the genus level and metabolites has an absolute Spearman rank correlation above 0.55 [gold line, positive correlation (*r* ≥ 0.55); blue line, negative correlation (*r* ≤ −0.55)] with a FDR-corrected significance level under 0.05. Microbes are shown by green round nodes, and metabolites are shown by pink diamond nodes.

*Anaerosporobacter* was positively associated (*r* > 0.63, *q* < 0.01) with phytanic acid and acetate, and was negatively associated (*r* < −0.57, *q* < 0.01) with 3-aminoisobutyric acid, propionate and butyrate (Figure 5). *Fibrobacter* was positively associated (*r* > 0.62, *q* < 0.01) with hydrocinnamic acid, phytanic acid, and acetate, and was negatively associated (*r* ≤ −0.55, *q* < 0.01) with 3-aminoisobutyric acid, NH_3_-N, and butyrate. *Succinimonas* was positively associated (*r* > 0.56, *q* < 0.02) with hydrocinnamic acid and acetate, and was negatively associated (*r* < −0.59, *q* < 0.01) with NH_3_-N and butyrate. *Saccharofermentans* was positively associated (*r* > 0.69, *q* < 0.01) with phytanic acid and acetate, and was negatively associated (*r* < −0.55, *q* < 0.02) with 3-aminoisobutyric acid and propionate. *Entodinium* was positively associated (*r* > 0.56, *q* < 0.01) with 3-aminoisobutyric acid and propionate, and was negatively associated (*r* < −0.70, *q* < 0.01) with acetate. *Ostracodinium* was positively associated (*r* > 0.69, *q* < 0.01) with acetate, and was negatively associated (*r* < −0.57, *q* < 0.01) with 3-aminoisobutyric acid and propionate.

## Discussion

In this study, the effects of wide F:C on ruminal microbiota and metabolites, as well as the interaction between them, were investigated. By definition, limit-feeding always results in decreasing DMI with increasing dietary concentrate levels (Zanton and Heinrichs, 2009, 2016). Even though rumen pH decreased linearly as there was a larger amount of fermentable carbohydrate in high-concentrate diets, the lowest pH value was more than 6.3, which indicated that all the animals had a healthy rumen internal environment (Plaizier et al., 2017). In contrast to previous studies, which focused on the injury caused by high-concentrate diets and low ruminal pH (Saleem et al., 2012; Mao et al., 2016), our study focused on the healthy microbial composition and nutritive metabolites other than bioamine or phenylacetate.

Our findings showed that even with the limit-feeding strategy, the highest concentrate diet (80%) still had a negative effect on bacterial richness (based on the Chao1 value) and diversity (based on the Shannon index), which was similar with 70% concentrate free-feeding cattle (Mao et al., 2013). *Bacteroidetes, Firmicutes*, and *Proteobacteria* were the most predominant bacteria in the rumen, which was recognized by most previous studies (Jami et al., 2013; Henderson et al., 2015; Shabat et al., 2016). *Prevotella, Succiniclasticum, Butyrivibrio, Ruminococcus, Fibrobacter*, and *Selenomonas* were both the core bacterial community and main bacteria in this study, which was similar to previous reports (Jami and Mizrahi, 2012; Li et al., 2012; Zhou et al., 2017). The higher abundance of these genera would enable their members to occupy various ecological niches and make full use of nutrients within the rumen (Jami and Mizrahi, 2012; Mao et al., 2015). Among them, *Butyrivibrio, Ruminococcus*, and *Fibrobacter* are known to be cellulolytic bacteria (Russell and Baldwin, 1978; Fernando et al., 2010; Zhou et al., 2017), whereas *Prevotella* is known for the utilization of starch and protein (Jami et al., 2013). *Succiniclasticum* and *Selenomonas* are specialized in fermenting succinate and converting it to propionate (van Gylswyk et al., 1997; Fernando et al., 2010; Shabat et al., 2016). The existence of these dominant bacteria was considered to be the result of the large amount of fiber (the mean neutral detergent fiber level was 31.1% dry matter; mostly from corn silage) and carbohydrate (the mean non-fibrous carbohydrate level was 42.8% dry matter; mostly from corn silage and steam-flaked corn) resources coexisting in these diets. Within the bacteria community that has been significantly changed by different dietary concentrate levels, *Acidithiobacillus, Christensenella*, and RFN54, had low relative abundances (<0.05%), revealing that their affects and functions might be minor.

In line with a number of previous reports (Sylvester et al., 2004; Kittelmann et al., 2013; Mao et al., 2016), *Entodinium* was the most abundant group in the rumen and linearly increased with increasing amounts of dietary concentrate in the current study. Mao et al. (2016) and Hook et al. (2011) also reported that the percentage of *Entodinium* that was able to engulf starch increased as the proportion of grain in diets increased. Similarly with a report from Kittelmann et al. (2013) that *Ostracodinium* was largely dominant in silage-fed ruminants (86% of the sequences obtained from cows fed silage), we found that *Ostracodinium* was the dominant genera in the rumen and linearly decreased with the increasing proportion of dietary concentrate level.

In the present study, the anaerobic fungi richness (based on Chao1 value) and diversity (based on Shannon index) might have some resistance to high-concentrate diets, which was similar with the report by Mao et al. (2016), while Kumar et al. (2015) found that high-concentrate (50%)-fed post-calving cows had lower Chao1 value and Shannon index (*P* < 0.05) than in low-concentrate (20%) fed cows. That difference might be due to transition cows having more sensitive rumen microbiota than heifers. Consistent with previous reports (Liggenstoffer et al., 2010; Koetschan et al., 2014; Mao et al., 2016), Neocallimastigomycota was the most predominant fungal phylum and linearly decreased as the proportion of dietary concentrate increased in this study, which might be explained by the important role of Neocallimastigomycota in the degradation of fibrous plant materials in ruminants (Gruninger et al., 2014; Koetschan et al., 2014). Several ruminal fungi that were rare in previous studies (Kittelmann et al., 2013; Mao et al., 2016), namely Ascomycota, Zygomycota, Basidiomycota, Cercozoa, and Chytridiomycota, were found in the current study. This result might be explained by the primers used for sequencing. In two previous studies (Kittelmann et al., 2013; Mao et al., 2016), the same primers were used and similar fungi taxa were observed; likewise, the present study used the same primers as reported by Kumar et al. (2015), as a consequence, similar fungal taxa were observed in these two studies. As these phyla were mostly detected from marine samples (Jones et al., 2015) and rarely found in herbivores, their metabolic and functional significance in the ruminal ecosystem is unknown. Therefore, the cause of the linear increase of Ascomycota, Basidiomycota, Cercozoa, and Chytridiomycota in high-concentrate-fed animals remains unclear.

The six known and predominant anaerobic fungi genera, namely *Anaeromyces, Caecomyces, Cyllamyces, Neocallimastix, Orpinomyces*, and *Piromyces*, were all obtained from this study. This finding was in accordance with most previous studies (Liggenstoffer et al., 2010; Gruninger et al., 2014; Kumar et al., 2015). *Anaeromyces, Cyllamyces, Neocallimastix, Orpinomyces*, and *Piromyces* have cellulase and/or xylanase activities (Liu et al., 2008; Kittelmann et al., 2012; Jin et al., 2014). *Neocallimastix* and *Orpinomyces* also have carbohydrate active enzymes (CAZYmes) or genes (Wang et al., 2011; Youssef et al., 2013). Therefore, the dominance of enzymes is likely dependent upon stimulation by the substrates provided. Kumar et al. (2015) reported that *Cyllamyces* remained unchanged between high (50%) and low (20%) concentration diets in primiparous cows, but the proportion of the genus *Cyllamyces* increased substantially (45%) in multiparous cows fed high-concentrate diets, while *Caecomyces* decreased in high-concentrate-diet fed cows, whether they were primiparous or multiparous (Kumar et al., 2015). Fernando et al. (2010) found *Orpinomyces* was abundant in cows fed a high-protein and hay-based diet. However, the coexistence of high-concentrate and high-forage diets among the four treatments might lead to a balance of the functions of cellulose and carbohydrate degradation, which resulted in the similar anaerobic fungi abundance at the genus level in the current study.

In this study, increasing levels of dietary concentrate decreased ruminal archaeal richness (based on Chao1 value), but had no significant effect on diversity (based on Shannon index), which was similar with results from Mao et al. (2016). In accordance with other studies (Kumar et al., 2015; Mao et al., 2016; Zhou et al., 2017), we also found that *Euryarchaeota* and *Methanobrevibacter* were the most abundant phylum and genus in the rumen, respectively. *Methanobrevibacter* was responsible for using hydrogen and/or formate to produce CH_4_ in ruminants (Danielsson et al., 2017). Increasing dietary concentrate levels did not result in a changing archaea community (Kumar et al., 2015; Mao et al., 2016), which is similar to this study. It has been reported that archaeal communities have less variation and diversity across diets and species than other microbial communities (Jeyanathan et al., 2011; Henderson et al., 2015). There might be two reasons for that. On one hand the total density of archaea was quite low (3–4% of total microbes) in the rumen, and on the other hand the archaea community might have the ability to be resilient to dietary changes (Kumar et al., 2015). Therefore, more work is needed to illuminate the abundance and function of archaea in the rumen.

In order to assess the variations of the rumen microbiome and metabolism in response to increasing limit-fed dietary concentrate, the trends or regression lines over the four gradients of concentrate, rather than performing between group comparisons, were investigated. Among the significantly affected metabolites in the present study, most of them were amino acids, such as 3-aminoisobutyric acid, alanine, and proline. Also, some aromatic compounds, like phenylacetic acid (Scott et al., 1964) and hydrocinnamic acid (Moss et al., 1970), were the degradation products of amino acids. This result was indicative that the increasing levels of dietary concentrate had significant effects on ruminal amino acid metabolism.

According to Saleem et al. (2013), approximately 55–60% of the rumen fluid metabolites were related to the rumen microbiota. We investigated the functional correlation between common significantly different metabolites and the changed rumen microbiome in the present study. Acetate, propionate, and butyrate were the main compositions of VFAs and the end-products of numbers of microbiota (Kamke et al., 2016). Among them, propionate was the major VFA contributing to gluconeogenesis (Young, 1977), and butyrate was found as a key factor stimulating the initial growth and differentiation of the rumen to become a mature absorptive organ (Ploger et al., 2012; Kamke et al., 2016). Therefore, the higher percentage of ruminal propionate and butyrate in groups fed higher-concentrate diets could be beneficial to the energy metabolism and development of the rumen in heifers, even though the detection of morphological features of ruminal epithelium cells should be warranted.

In the process of synthesizing acetate, propionate, and butyrate, pyruvate was a competitive substrate for them, and the butyrate level could be raised as well as a higher propionate content occurring (Russell and Rychlik, 2001; Sutton et al., 2003; Ploger et al., 2012). As *Anaerosporobacter, Saccharofermentans*, and *Ostracodinium* produced acetate as main end-products (Petzel and Hartman, 1990; Ziemer, 2014; Ziganshina et al., 2014), it could be partly explained that these three bacteria were found to be negatively associated with propionate and butyrate, while positively associated with acetate in the present study (Figure 5). *Entodinium* contributed a lot to propionate production in the rumen, which might be due to *Entodinium* being a starch degrader (Ivan et al., 2000). As acetate also was one of main end-products of *Fibrobacter* and *Succinimonas* or their symbiotic microbes (Forano et al., 2008), that could be the reason that acetate was positively related with these two bacteria in this study (*r* > 0.60, *q* < 0.01).

As NH_3_-N is one essential nitrogen source for *Fibrobacter* (Atasoglu et al., 2001), it is easy to understand the negative relationship between *Fibrobacter* and NH_3_-N that was found in this study (*r* < −0.71, *q* < 0.01). Since *Fibrobacter* and *Succinimonas* prefer to utilize fiber in high-forage diets and were less prevalent in high-concentrate diets in the present study, NH_3_-N was also negatively associated with *Succinimonas* (Figure 5). The NH_3_-N concentration in the C80 group was higher than 5 mg/dL (Table 1), which was the minimum requirement for maximal microbial growth (Satter and Slyter, 1974); this was indicative that a high-concentrate diet could produce more ruminal microbe crude protein for utilization by the animals.

In ruminants, proline, which can be formed by arginine and glutamine/glutamate, was found to be one of the most abundant (approximately 12%) amino acids in casein and played important roles in protein synthesis, structure, metabolism, and nutrition (Bruckental and Alumot, 1984; Wu et al., 2011). When more proline was added into the diet, approximately 20% of arginine was saved (Alumot et al., 1983). Therefore, higher ruminal proline resulting from feeding high-concentrate diets could be beneficial to ruminal amino acid metabolism and whole-body protein synthesis. Also, proline was found to have the ability to enhance the digestion of cellulose by rumen microorganisms (Dehority et al., 1958; Amos et al., 1971). The fact that proline stimulates *in vitro* rumen microbial cellulose digestion may be explained partially by its conversion to valeric acid (Amos et al., 1971). In this study, proline was negatively associated with *Fibrobacter* (*r* ≤ −0.55, *q* < 0.02), which was suggestive that less proline was degraded in the rumen of heifers fed high-concentrate diets.

Among amino acids, peptides, and analogs, 3-aminoisobutyric acid was an intermediate of valine degradation in the pathways of valine, leucine, and isoleucine degradation (Podebrad et al., 2000). Valine was firstly degraded to isobutyryl-CoA and then to 3-aminoisobutyric acid, while branched chain fatty acid was also formed by isobutyryl-CoA (KEGG, Map 00280). Therefore, 3-aminoisobutyric acid and branched chain fatty acid were competitive in the rumen. Dehority et al. (1958) reported that valine could be oxidatively deaminated and decarboxylated by rumen microorganisms to form isobutyrate, and that isobutyrate had been shown to enhance the digestion of cellulose by rumen microorganisms *in vitro*. In the current study, 3-aminoisobutyric acid was negatively associated (*r* < −0.58, *q* < 0.05) with cellulolytic bacteria (such as *Fibrobacter* and *Anaerosporobacter*), as well as isobutyrate quadratically decreasing with increasing dietary concentrate levels (Table 1), which could be explained by most valine being degraded to isobutyryl-CoA then forming 3-aminoisobutyric acid other than isobutyrate in the rumen of high-concentrate-fed heifers.

Even though hydrocinnamate can be derived from phenylalanine (Moss et al., 1970), lactic acid bacteria have the enzymatic ability to convert cinnamate into hydrocinnamate (Broberg et al., 2007). The concentration of hydrocinnamate was found to be substantially higher in silage inoculated with lactic acid bacteria (Broberg et al., 2007). In the present study, the lower amount of corn silage (from 80 to 20% dry matter in diets, Table S1) used in high-concentrate diets could explain the linear decrease of hydrocinnamate in the rumen as the content of dietary concentrate was elevated, which was in line with work by Saleem et al. (2012). It was reported that hydrocinnamate could stimulate the growth of *Ruminococcus albus* 7, *Ruminococcus flavefaciens* FD-1, *Butyrivibrio fibrisolvens* 49, and *Lachnospira multipara* D-32, while the stimulation of the cellulolytic activity of these bacteria had species specificity (Borneman et al., 1986). This might be indicative that some species of *Fibrobacter* and *Succinimonas* could be specifically stimulated by hydrocinnamate in the current study.

Phytanic acid originates from chlorophylls (Dawson et al., 1974). One of the intermediate products, dihydrophytol, was hydrogenated and oxidized by rumen microorganisms to form phytanic acid, which led to a slow accumulation of phytanic acid in the ruminant's body and products (Hansen, 1966; Dawson et al., 1974). Hansen (1966) found that phytanic acid could account for 2.9% of the total fatty acids from the rumen bacteria of dairy cows fed a clover-grass hay diet. The bacteria, such as *Anaerosporobacter, Fibrobacter, Saccharofermentans*, and *Anaeroplasma*, that had the ability to degrade cellulose could release more chlorophyll and form phytanic acid in the present study.

In summary, the present study combined microbiome and metabolomics analysis to investigate the effects of limit-feeding four increasing gradient levels of dietary concentrate on ruminal microbial communities and metabolites under healthy conditions. With the increasing amount of dietary concentrate, the cellulolytic related bacteria and ciliate protozoa largely decreased, while other microbiota remained stable or slightly changed. Utilization or productive association was widely found between affected microbiota and certain metabolites. This study has allowed a better understanding of ruminal metabolites and microbial functions, which could further provide integrative information about rumen function and lead to improvements in ruminant production, such as increased digestion and feed efficiency.

## Author contributions

YW, SL, ZC, JZ, and HS conceived and designed the experiments. HS, SJ, HZ, and JZ conducted the experiments. JZ, HS, SJ, and HZ performed the statistical analysis of the experimental data. Finally, the paper was written by JZ and HS, and was modified by YW and YH. All authors read and approved the final manuscript.

### Conflict of interest statement

The authors declare that the research was conducted in the absence of any commercial or financial relationships that could be construed as a potential conflict of interest.

## Supplementary material

Figure S1 Venn diagram illustrating overlap of microbial OTUs at 3% dissimilarity level among treatments. Venn diagram of bacteria **(A)**, ciliate protozoa **(B)**, anaerobic fungi **(C)**, and archaea **(D)** OTUs. C20, diet containing 20% of concentrate; C40, diet containing 40% of concentrate; C60, diet containing 60% of concentrate; C80, diet containing 80% of concentrate.

Figure S2 Effect of increasing concentrate diets on changes of phyla. **(A)** Effect of increasing concentrate diets on changes in the top 15 most abundant phyla of bacteria. **(B)** Effect of increasing concentrate diets on changes in the most abundant phyla of anaerobic fungi. C20, diet contained 20% of concentrate; C40, diet contained 40% of concentrate; C60, diet contained 60% of concentrate; C80, diet contained 80% of concentrate.

Figure S3 Increasing dietary concentrate altered predominant microbial taxa (as a percentage of the total sequences). **(A)** Distribution of the top 15 (mean relative abundance) bacterial genera across the dietary treatments. **(B)** Distribution of the predominant (accounting for over 1.0% of the total sequences in at least one of the samples) ciliate protozoa taxa averaged across the dietary treatments. **(C)** Distribution of the top 15 (mean relative abundance) anaerobic fungi genera averaged across the dietary treatments. **(D)** Distribution of the archaea taxa across the dietary treatments. C20, diet containing 20% of concentrate; C40, diet containing 40% of concentrate; C60, diet containing 60% of concentrate; C80, diet containing 80% of concentrate.

Figure S4 High-resolution GC-TOF/MS total ion chromatograms (TIC) of the rumen samples corresponding to different diets. GC-TOF/MS TIC chromatograms of rumen samples for cows fed 20% **(A)**, 40% **(B)**, 60% **(C)**, and 80% **(D)** concentrate.

Figure S5 Total PCA plot of the rumen samples corresponding to different diets. C20, diet contained 20% of concentrate; C40, diet contained 40% of concentrate; C60, diet contained 60% of concentrate; C80, diet contained 80% of concentrate; QC, quality control.

Table S2 Basic information of identified metabolites. C20, diet contained 20% of concentrate; C40, diet contained 40% of concentrate; C60, diet contained 60% of concentrate; C80, diet contained 80% of concentrate.

## Abbreviations

AMOVA: analysis of molecular variance
DMI: dry matter intake
F:C: forage to concentrate ratios
FDR: false discovery rate
GC-TOF/MS: gas chromatography time-of-fight/mass spectrometry
OPLS-DA: orthogonal projections to latent structures-discriminant analysis
OTU: operational taxonomic unit
PCA: principal component analysis
PCoA: principal coordinate analysis
VFA: volatile fatty acid
VIP: variable importance projection.
