# Supplementary figures and images for: Effect of Dietary Forage to Concentrate Ratios on Dynamic Profile Changes and Interactions of Ruminal Microbiota and Metabolites in Holstein Heifers
Source: Front Microbiol. 2017 Nov 9;8:2206. doi: 10.3389/fmicb.2017.02206 (PMC5684179; doi:10.3389/fmicb.2017.02206)

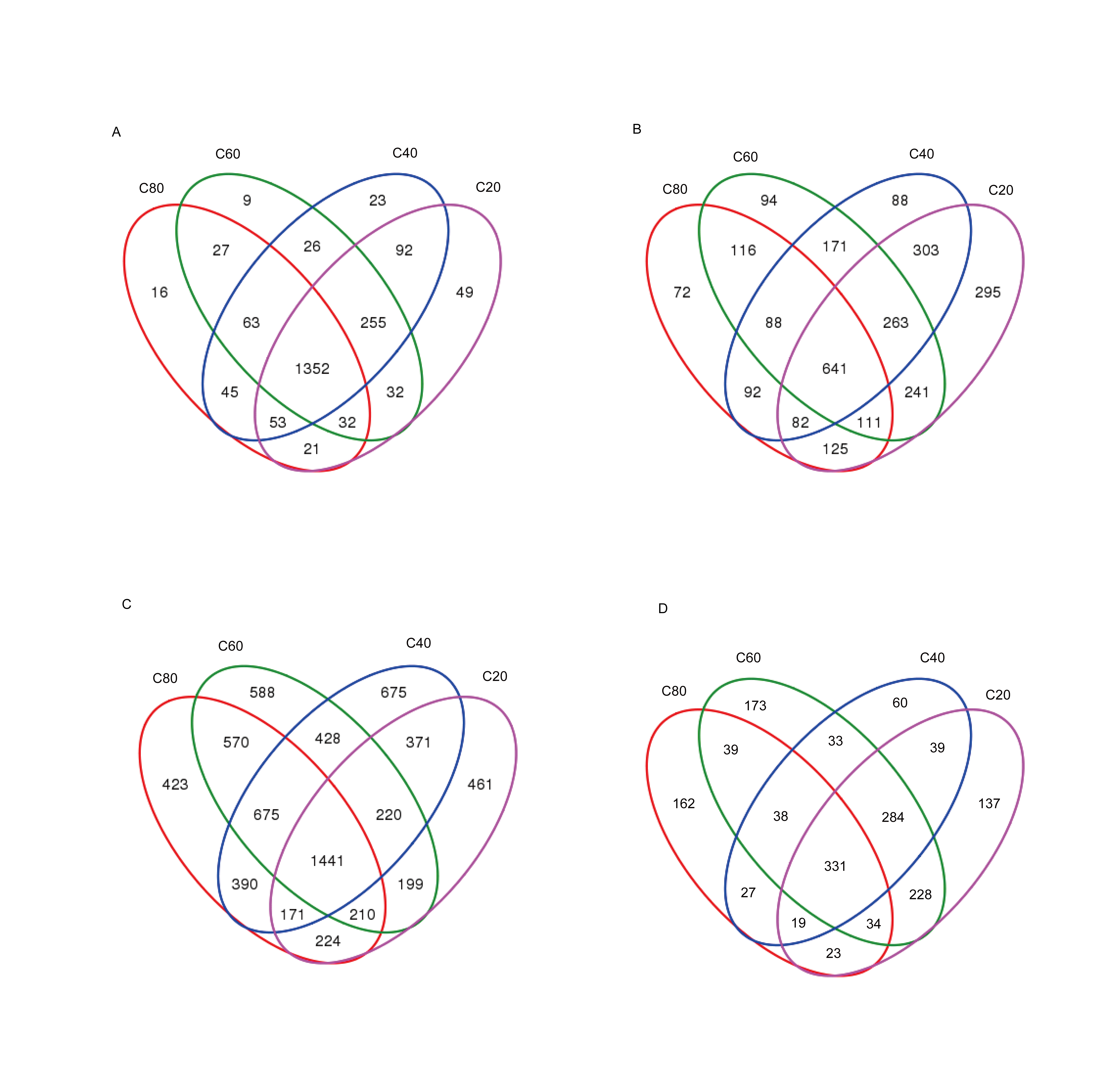

Supplement: Figure S1 — Venn diagram illustrating overlap of microbial OTUs at 3% dissimilarity level among treatments. Venn diagram of bacteria (A), ciliate protozoa (B), anaerobic fungi (C), and archaea (D) OTUs. C20, diet containing 20% of concentrate; C40, diet containing 40% of concentrate; C60, diet containing 60% of concentrate; C80, diet containing 80% of concentrate. [file Image1.TIF]

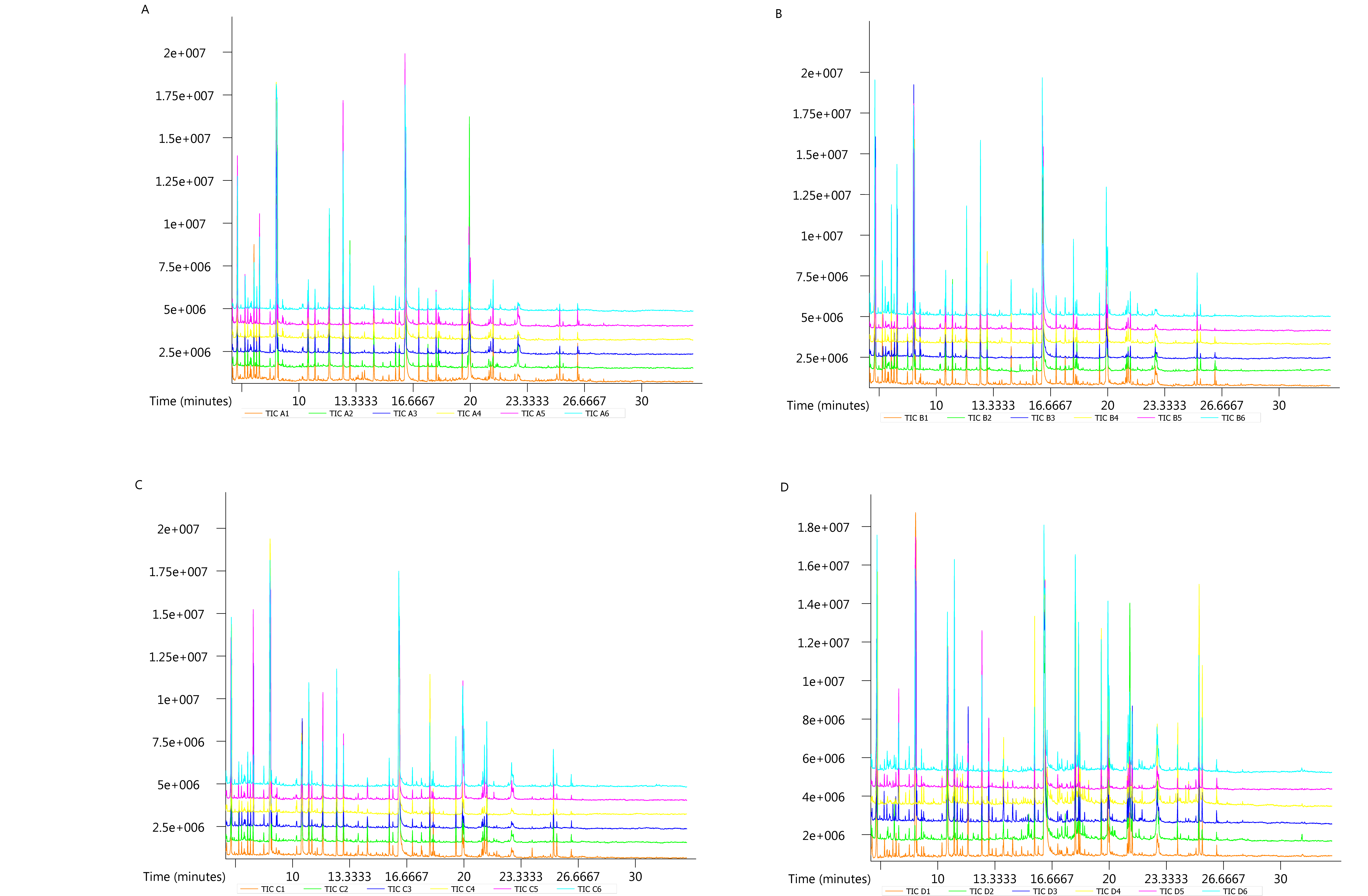

Supplement: Figure S4 — High-resolution GC-TOF/MS total ion chromatograms (TIC) of the rumen samples corresponding to different diets. GC-TOF/MS TIC chromatograms of rumen samples for cows fed 20% (A), 40% (B), 60% (C), and 80% (D) concentrate. [file Image4.TIF]

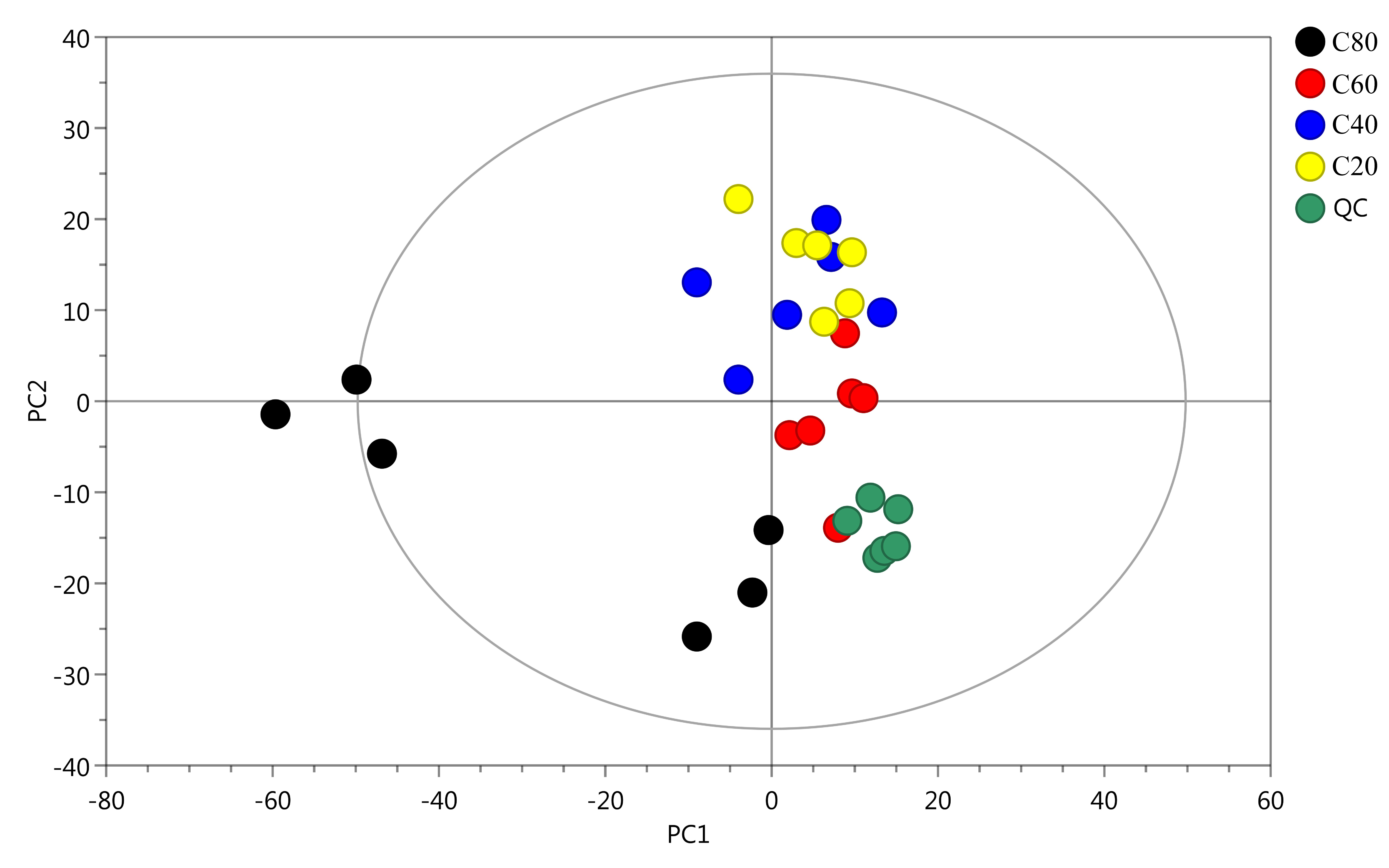

Supplement: Figure S5 — Total PCA plot of the rumen samples corresponding to different diets. C20, diet contained 20% of concentrate; C40, diet contained 40% of concentrate; C60, diet contained 60% of concentrate; C80, diet contained 80% of concentrate; QC, quality control. [file Image5.TIF]
